# Hidden in Plain Sight: Gonorrhea‐Associated Flexor Tenosynovitis—A Case Report

**DOI:** 10.1155/crdi/8215577

**Published:** 2026-10-26

**Authors:** Jason Chavez, Joseph Aguilar, Madiha Ahmed, Shaoli Chaudhuri

**Affiliations:** ^1^ Department of Infectious Diseases Jackson Memorial Hospital Miami Florida USA jhsmiami.org; ^2^ Department of Internal Medicine Mt. Sinai Medical Center Miami Beach Florida USA

**Keywords:** case report, disseminated gonococcal infection, flexor tenosynovitis, gonococcal tenosynovitis, *Neisseria gonorrhoeae*

## Abstract

Disseminated gonococcal infection (DGI) is an uncommon but potentially serious complication of *Neisseria gonorrhoeae* infection in which infection extends from a mucosal site to distant tissues. The literature describes two types of clinical presentation: an arthritis–dermatitis syndrome characterized by tenosynovitis, polyarthralgia, and skin lesions and a suppurative monoarthritis. In the former presentation, synovial fluid cultures are usually negative, whereas synovial fluid cultures are more often positive in the latter suppurative gonococcal arthritis. This distinction is important in the present case because *Neisseria gonorrhoeae* was recovered from the surgically drained collection associated with the fourth‐digit flexor tendon sheath rather than from synovial fluid; therefore, we characterize the case as gonococcal flexor tenosynovitis associated with DGI rather than culture‐proven septic arthritis. A 53‐year‐old man with well‐controlled HIV presented with pain, swelling, and limited motion of the right fourth finger and shoulder after recent travel to Cuba and sexual contact with a new male partner with inconsistent condom use. Laboratory evaluation revealed leukocytosis and elevated inflammatory markers. Ultrasound of the finger demonstrated flexor tenosynovitis with a 10‐mm abscess. Bedside incision and drainage was performed, and the surgical specimen grew *Neisseria gonorrhoeae*. The patient was treated with intravenous ceftriaxone followed by oral amoxicillin based on antimicrobial susceptibility results, with complete clinical recovery. This case highlights an unusual localized musculoskeletal manifestation of DGI in the absence of urogenital symptoms. Point‐of‐care ultrasound identified a drainable collection, while culture of the affected site established the diagnosis. DGI should be considered in atraumatic flexor tenosynovitis, particularly when there is a relevant sexual exposure.

## 1. Introduction

Disseminated gonococcal infection (DGI) is an uncommon complication of *Neisseria gonorrhoeae* infection in which infection extends from a mucosal site to distant tissues. Two major clinical patterns have been described: the first consists of an arthritis–dermatitis syndrome characterized by tenosynovitis, polyarthralgia, and skin lesions, and the second consists of a suppurative monoarthritis. A distinguishing factor is that cultures tend to be negative in the former and positive in the latter [1, 2]. This distinction is important in the present case as *Neisseria gonorrhoeae* was recovered from the surgically drained collection associated with the fourth‐digit flexor tendon sheath rather than from synovial fluid; therefore, we characterize the case as gonococcal flexor tenosynovitis (FTS) associated with DGI rather than culture‐proven septic arthritis.

Tenosynovitis is a recognized manifestation of DGI and may involve the flexor tendon sheath [1, 3, 4]. Physical examination findings may include Kanavel’s signs—tenderness over the tendon sheath, fusiform swelling, flexed posture, and pain with passive extension—in a sexually active patient without a history of trauma [5]. Diagnosis requires clinical suspicion together with microbiologic evaluation of appropriate mucosal and disseminated sites. Point‐of‐care ultrasound (POCUS) may identify a complex hypoechoic or anechoic fluid collection surrounding the tendon and can help identify an abscess [6].

Antimicrobial therapy is the cornerstone of treatment for DGI, while surgical consultation and drainage or debridement may be appropriate when there is a purulent collection, abscess, or persistent local infection [2, 7]. Treatment should be guided by susceptibility data when an isolate is recovered. Patients should also receive counseling regarding safer sex practices and evaluation and treatment of sexual partners as indicated [8].

In this report, we present an unusual case of right fourth‐digit flexor tendon tenosynovitis associated with DGI and shoulder involvement in the absence of urogenital symptoms.

## 2. Case Presentation

A 53‐year‐old man with a history of well‐controlled HIV infection (last CD4 count: 1350 cells/mm^3^, undetectable viral load) presented to the emergency department with 4 days of pain in his right fourth finger and shoulder. The patient had no prior history of hand trauma but reported subjective fever, chills, headache, malaise, and diarrhea 1 week earlier during a trip to Cuba. He reported sexual activity with a new male partner during the trip, with inconsistent condom use and no reported urogenital symptoms.

On examination, the patient was afebrile. The right fourth digit was swollen, tender, and erythematous (Figure 1), with limited range of motion in the fourth digit and shoulder.

**FIGURE 1:**
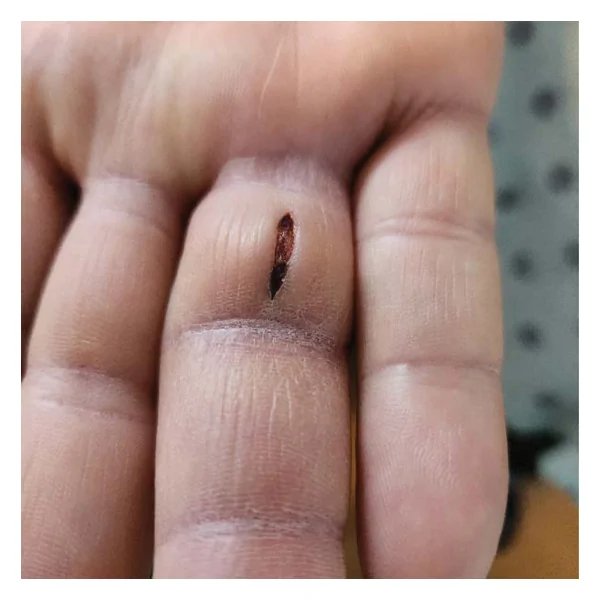
Right fourth digit demonstrating localized swelling and erythema associated with flexor tenosynovitis. The appearance represents the involved digit and should not be interpreted as a spontaneous gonococcal skin ulcer.

Laboratory evaluation revealed leukocytosis (13.6 × 10^3^/mcL) and elevated C‐reactive protein (5.3 mg/dL) and erythrocyte sedimentation rate (35 mm/h). Blood cultures showed no growth, and urinary gonorrhea and chlamydia nucleic acid amplification tests (NAATs) were negative. Oral/pharyngeal and rectal gonorrhea/chlamydia swabs were collected but were not processed.

Right‐hand radiography was unremarkable; however, ultrasound (US) of the right fourth digit revealed a 5 × 10 mm hypoechoic, well‐circumscribed region superficial to the tendon sheath with adjacent mild soft tissue edema, consistent with a localized fluid collection.

The patient was diagnosed with FTS of the fourth finger with an associated 10‐mm abscess. The orthopedic surgery team was consulted, and he underwent bedside incision and drainage of the right fourth finger because of the localized drainable collection.

Following the procedure, the patient was empirically started on intravenous cefepime 2 g every 8 h and vancomycin, with dosing managed by the pharmacy. Surgical cultures identified *Neisseria gonorrhoeae*, and Infectious Diseases was consulted. The patient was switched to intravenous ceftriaxone 2 g daily based on susceptibility results (Table 1). By Day 5 of hospitalization, his symptoms had almost completely resolved, and he was discharged to complete 1 week of ceftriaxone 2 g daily, followed by a 1‐week course of oral amoxicillin 1 g three times daily based on the isolate’s susceptibility. The patient was educated on safer sex practices and encouraged to refer his sexual partners for screening and treatment as appropriate.

**TABLE 1:** Antimicrobial susceptibility testing of the *Neisseria gonorrhoeae* isolate recovered from the patient’s right fourth‐digit surgical specimen.

Antibiotic	Interpretation	MIC (*μ*g/mL)
Azithromycin	Susceptible	0.5
Ceftriaxone	Susceptible	< 0.002
Ciprofloxacin	Nonsusceptible	1
Ertapenem	No CLSI breakpoint	0.006
Meropenem	No CLSI breakpoint	0.008
Penicillin	Susceptible	0.047
Trimethoprim–sulfamethoxazole	No CLSI breakpoint	4

*Note:* “No CLSI breakpoint” indicates that no CLSI interpretive breakpoint was established for the corresponding antimicrobial agent.

Abbreviations: CLSI = Clinical and Laboratory Standards Institute, MIC = minimum inhibitory concentration.

At 1‐week follow‐up after discharge, the patient was adherent to his oral antibiotic regimen and had complete resolution of his symptoms (Table 2). No adverse events were reported during treatment or follow‐up.

**TABLE 2:** Timeline of events from presentation to postdischarge follow up.

Time	Clinical event
Approximately 1 week before presentation	During a trip to Cuba, the patient experienced subjective fever, chills, headache, malaise, and diarrhea and reported sexual activity with a new male partner with inconsistent condom use
Day of admission (Day 1)	Presented with 4 days of right fourth‐finger and shoulder pain. He was afebrile; the right fourth digit was swollen, tender, and erythematous with limited range of motion
Day of admission (Day 1)	Laboratory testing showed WBC 13.6 × 10^3^/mcL, CRP 5.3 mg/dL, and ESR 35 mm/h. Blood cultures and urinary gonorrhea/chlamydia NAAT were negative. Extragenital swabs were collected but not processed
Hospital day 2	Ultrasound demonstrated a 5 × 10 mm fluid collection adjacent to the right fourth‐digit tendon sheath
Hospital day 3	Bedside incision and drainage of the right fourth finger was performed by orthopedic surgery. Empiric cefepime and vancomycin were started.
Hospital day 4	Antibiotics were changed to intravenous ceftriaxone based on susceptibility results
Hospital day 5	Symptoms had almost completely resolved. The patient was discharged to complete 1 week of ceftriaxone followed by 1 week of oral amoxicillin 1 g three times daily
1 week after discharge	Complete resolution of symptoms was documented at follow‐up

## 3. Discussion

The patient presented with an unusual localized musculoskeletal manifestation of DGI involving the right fourth‐digit flexor tendon sheath, with concurrent shoulder symptoms. His relevant risk factors included HIV infection, sex with men, and inconsistent use of barrier methods. DGI is classically described as either an arthritis–dermatitis syndrome with tenosynovitis and polyarthralgia or suppurative monoarthritis [1, 2]. These have been described as distinct clinical groups, with synovial fluid cultures frequently positive in the suppurative arthritis group, whereas tenosynovitis and skin manifestations tend to be culture‐negative [1]. Our patient did not have culture‐proven joint infection because no synovial fluid was obtained; instead, *Neisseria gonorrhoeae* was recovered from the surgically drained collection associated with the tendon sheath. This distinction supports describing the case as gonococcal FTS rather than septic gonococcal arthritis.

The physical examination demonstrated Kanavel’s signs—tenderness over the tendon sheath, fusiform swelling, flexed posture, and pain with passive extension—which can support the diagnosis of flexor sheath infection [5]. The absence of urogenital symptoms and a negative urinary NAAT further illustrate that a localized mucosal infection may not be clinically apparent. Contemporary epidemiologic work also emphasizes that DGI is uncommon and that the factors associated with dissemination are incompletely understood [9–11].

This case also illustrates the value of bedside US in identifying a drainable collection in a patient whose presentation could otherwise resemble routine hand infection. The failure to process the oral, pharyngeal, and rectal specimens represents an important diagnostic limitation because it prevented identification of the likely mucosal site of acquisition.

Strengths of this case include microbiologic confirmation of *Neisseria gonorrhoeae* from the affected surgical site, detailed antimicrobial susceptibility testing, use of POCUS to identify a localized collection, and documented clinical resolution after treatment. Limitations include negative blood and urinary testing, lack of processed extragenital specimens, absence of synovial fluid testing, and the inability to determine the primary mucosal site or characterize the infecting strain. These limitations also make it difficult to establish whether the patient represented the classic bacteremic arthritis–dermatitis phenotype or a localized gonococcal infection with musculoskeletal dissemination.

Antimicrobial therapy is the cornerstone of treatment for DGI, and many patients respond rapidly and completely to antibiotics alone [2, 7]. Surgical intervention should therefore be individualized rather than considered mandatory for gonococcal tenosynovitis or arthritis. In this case, bedside incision and drainage was performed because US demonstrated a discrete 10‐mm abscess associated with the flexor tendon sheath, providing both source control and a diagnostic specimen. Once *Neisseria gonorrhoeae* was identified, ceftriaxone was selected based on susceptibility results. The patient’s isolate was susceptible to penicillin, which informed the subsequent use of oral amoxicillin to complete therapy.

The susceptibility profile is also consistent with the observation that gonococcal strains associated with dissemination have historically shown high antimicrobial susceptibility, including susceptibility to penicillin. Recent discussion of the epidemiology of DGI has revisited the possible role of historical arginine, hypoxanthine, and uracil (AHU) auxotypes and their antimicrobial susceptibility patterns, although the relevance of these historical observations to contemporary DGI remains uncertain [9–11]. Our isolate was susceptible to penicillin and ceftriaxone, while ciprofloxacin was reported as nonsusceptible. The susceptibility results are therefore clinically relevant to the individualized antibiotic selection in this case, but they should not be generalized to current empiric treatment of gonococcal infection.

Given the variable presentation of DGI and the potential for musculoskeletal disease to mimic other infections, clinicians should consider gonococcal infection in sexually active patients with atraumatic tenosynovitis, particularly when there is a compatible systemic prodrome or relevant sexual exposure. Early microbiologic sampling, appropriate antimicrobial therapy, and selective surgical management when a drainable collection is present may help achieve favorable outcomes.

## 4. Conclusion

DGI can present with an unusual localized musculoskeletal manifestation such as culture‐confirmed gonococcal FTS without urogenital symptoms. A careful sexual history, consideration of DGI in atraumatic tenosynovitis, and targeted microbiologic testing can facilitate diagnosis. POCUS may rapidly identify a drainable collection, while antimicrobial therapy remains the cornerstone of treatment and surgical drainage should be reserved for appropriate source‐control indications. In this patient, susceptibility‐guided therapy and drainage of the localized abscess were associated with complete clinical recovery.

## Author Contributions

Jason Chavez and Joseph Aguilar drafted the initial case report. Madiha Ahmed composed the abstract and revised the manuscript. Shaoli Chaudhuri supervised the case report writing process, provided relevant feedback, and approved the manuscript for submission.

## Funding

This manuscript received no external funding.

## Disclosure

All authors reviewed and approved the final manuscript.

## Ethics Statement

Written informed consent was obtained from the patient for publication of this case report and any accompanying clinical images. Institutional Review Board approval was not required for this case report in accordance with institutional policies.

## Conflicts of Interest

The authors declare no conflicts of interest.

## Supporting Information

Additional supporting information can be found online in the Supporting Information section.

## Supporting information


**Supporting Information** DGI care checklist.

## Data Availability

The data supporting the findings of this case report are available from the corresponding author upon reasonable request, subject to applicable privacy and institutional requirements.
