# Supplementary material for: Hidden in Plain Sight: Gonorrhea‐Associated Flexor Tenosynovitis—A Case Report
Source: Case Rep Infect Dis. 2026 Oct 26;2026:8215577. doi: 10.1155/crdi/8215577 (PMC13698652; doi:10.1155/crdi/8215577)
Supplement: Supplementary file 1 [file CRDI-2026-8215577-s001.pdf]

CARE case report guidelines

CARE Checklist of information to include when writing a case report

(cc) BY-NC-ND

Topic	Item	Checklist item description	Reported on Line
Title	1	The diagnosis or intervention of primary focus followed by the words “case report”	1
Key Words	2	2 to 5 key words that identify diagnoses or interventions in this case report, including "case report"	9,10
Abstract (no references)	3a	Introduction: What is unique about this case and what does it add to the scientific literature?	38-47
	3b	Main symptoms and/or important clinical findings	64-68
	3c	The main diagnoses, therapeutic interventions, and outcomes	82, 84, 86-92, 95-97
	3d	Conclusion—What is the main “take-away” lesson(s) from this case?	165-172
Introduction	4	One or two paragraphs summarizing why this case is unique (**may include references**)	110-117
Patient Information	5a	De-identified patient specific information.	63-65
	5b	Primary concerns and symptoms of the patient.	65-67
	5c	Medical, family, and psycho-social history including relevant genetic information	63
	5d	Relevant past interventions with outcomes	N/A
Clinical Findings	6	Describe significant physical examination (PE) and important clinical findings.	69-73
Timeline	7	Historical and current information from this episode of care organized as a timeline	98
Diagnostic Assessment	8a	Diagnostic testing (such as PE, laboratory testing, imaging, surveys).	71-78
	8b	Diagnostic challenges (such as access to testing, financial, or cultural)	77-78
	8c	Diagnosis (including other diagnoses considered)	82-83
	8d	Prognosis (such as staging in oncology) where applicable	138-139
Therapeutic Intervention	9a	Types of therapeutic intervention (such as pharmacologic, surgical, preventive, self-care)	84, 86-92
	9b	Administration of therapeutic intervention (such as dosage, strength, duration)	86-92
	9c	Changes in therapeutic intervention (with rationale)	91-92
Follow-up and Outcomes	10a	Clinician and patient-assessed outcomes (if available)	95-97
	10b	Important follow-up diagnostic and other test results	80, 87-88, 95-97
	10c	Intervention adherence and tolerability (How was this assessed?)	95-97
	10d	Adverse and unanticipated events	96
Discussion	11a	A scientific discussion of the strengths AND limitations associated with this case report	130-137
	11b	Discussion of the relevant medical literature **with references**.	38-47, 55-57, 122-124
	11c	The scientific rationale for any conclusions (including assessment of possible causes)	113-117
	11d	The primary “take-away” lessons of this case report (without references) in a one paragraph conclusion	165-172
Patient Perspective	12	The patient should share their perspective in one to two paragraphs on the treatment(s) they received	N/A
Informed Consent	13	Did the patient give informed consent? Please provide if requested	Yes ☑ No ☐
